# Is mean heart dose a relevant surrogate parameter of left ventricle and coronary arteries exposure during breast cancer radiotherapy: a dosimetric evaluation based on individually-determined radiation dose (BACCARAT study)

**DOI:** 10.1186/s13014-019-1234-z

**Published:** 2019-02-07

**Authors:** Sophie Jacob, Jérémy Camilleri, Sylvie Derreumaux, Valentin Walker, Olivier Lairez, Mathieu Lapeyre, Eric Bruguière, Atul Pathak, Marie-Odile Bernier, Dominique Laurier, Jean Ferrieres, Olivier Gallocher, Igor Latorzeff, Baptiste Pinel, Denis Franck, Christian Chevelle, Gaëlle Jimenez, David Broggio

**Affiliations:** 1 Institut de Radioprotection et de Sureté Nucléaire (IRSN), PSE-SANTE, SESANE, LEPID, BP17, 92262 Fontenay-aux-roses, Cedex France; 2 Clinique Pasteur, Radiothérapie (Oncorad), Toulouse, France; 3 Institut de Radioprotection et de Sureté Nucléaire (IRSN), PSE-SANTE, SER, UEM, Fontenay-aux-Roses, France; 4 University Hospital Rangueil, Cardiac Imaging Center, Toulouse, France; 5 Clinique Pasteur, Radiologie, Toulouse, France; 6 Clinique Pasteur, Unité d’Hypertension Artérielle, Facteurs de Risque et Insuffisance Cardiaque, Toulouse, France; 7 Institut de Radioprotection et de Sureté Nucléaire (IRSN), PSE-SANTE, SESANE, Fontenay-aux-Roses, France; 8 Department of Cardiology, Toulouse-Rangueil University Hospital, Toulouse, France; 9 INSERM, University Paul Sabatier, UMR1027, Epidemiology of Cardiovascular Diseases, Toulouse, France; 10 Institut de Radioprotection et de Sureté Nucléaire (IRSN), PRP-HOM/SDOS/LEDI, Fontenay-aux-Roses, France

**Keywords:** Breast cancer, Radiotherapy, Heterogeneity in cardiac exposure, Mean heart dose, Coronary arteries doses, Cardiotoxicity

## Abstract

**Background:**

Intra-individual heterogeneity of cardiac exposure is an issue in breast cancer (BC) radiotherapy that was poorly considered in previous cardiotoxicity studies mainly based on mean heart dose (MHD). This dosimetric study analyzes the distribution of individually-determined radiation doses to the heart and its substructures including coronary arteries and evaluate whether MHD is a relevant surrogate parameter of dose for these substructures.

**Methods:**

Data were collected from the BACCARAT prospective study that included left or right unilateral BC patients treated with 3D-Conformal Radiotherapy (RT) between 2015 and 2017 and followed-up for 2 years with repeated cardiac imaging examinations. A coronary computed tomography angiography (CCTA) was performed before RT for all patients. Registration of the planning CT and CCTA images allowed delineation of the coronary arteries on the planning CT images. Using the 3D dose matrix generated during treatment planning and the added coronary contours, dose distributions were generated for whole heart and the following substructures: left ventricle (LV), left main coronary artery (LMCA), left anterior descending artery (LAD), left circumflex artery (LCX) and right coronary artery (RCA). A descriptive analysis of the physical doses in Gray (Gy) was performed, Dmean was the volume-weighted mean dose.

**Results:**

Dose distributions were generated for 89 left-sided BC patients (MHD = 2.9 ± 1.5 Gy, Dmean_LAD = 15.7 ± 3.1 Gy) and 15 right-sided BC patients (MHD = 0.5 ± 0.1 Gy; Dmean_RCA = 1.2 ± 0.4 Gy). For left-sided BC patients, the ratio Dmean_LAD/MHD was around 5. Pearson correlation coefficients between MHD and Dmean for delineated substructures were all statistically significant. However, for all substructures, the coefficient of determination R^2^ indicated that the proportion of the variance in Dmean of the substructure predictable from MHD was moderate to low (in particular R^2^ = 0.45 for LAD). Among left-sided BC patients with MHD < 3Gy, 56% of patients could nevertheless receive LAD doses above 40Gy (V40 > 0).

**Conclusion:**

Our study illustrates that MHD is not enough to predict with confidence individual patient dose to the LV and coronary arteries, in particular the LAD. For precise radiotherapy-induced cardiotoxicity studies it would be necessary to consider the distribution of doses within these cardiac substructures rather than just the MHD.

**Trial registration:**

ClinicalTrials.gov: NCT02605512, Registered 6 November 2015 - Retrospectively registered.

## Introduction

Radiotherapy (RT) for breast cancer is an essential part of adjuvant cancer treatment. RT reduces the risk of local recurrence and the risk of breast cancer mortality [1].

However, left-sided RT, especially, has been shown to induce excess cardiovascular mortality and morbidity [2–6]. The study by Darby et al. [5] found a linear relationship between the mean heart dose (MHD) and the rate of major coronary event, which increased of 7.4% per Gy of the MHD. These results on radiation-induced ischemic heart disease were confirmed in a more recent study of BC patients treated with three dimensional conformal radiation therapy (3D-CRT) [7]. The predominance of ischemic heart disease observed in these studies indicates that the coronary arteries may be the critical structures for the development of radiation induced heart morbidity. Among the three major coronary arteries (left anterior descending, circumflex, and right), the left anterior descending (LAD) supplies a major part of the myocardium. Therefore, occlusion of the LAD, may cause a large area of myocardial necrosis and lead to severe left ventricle impairment and congestive heart failure.

However, in many epidemiological studies on post radiotherapy cardiotoxicity, doses are typically described as those received by the entire heart with the MHD [5, 8] . Therefore, the MHD is used as the reference dose for analyzing dose-response relationship. But, dose distribution in the heart is not homogeneous, highest cardiac radiation doses can be observed in the apex and in the apical-anterior segment and some hot spots>50Gy persist in some parts of the heart [9]. Nevertheless, the anatomic distribution of RT-associated coronary artery diseases has been poorly studied whereas it can be supposed that the increased risk of coronary artery diseases would manifest largely in the coronary arteries that are directly within the radiation field. This was confirmed by a Sweden study [10] where patients with left-sided breast cancer RT had a statistically significant increased rate of stenosis in the LAD when compared with those with right-sided cancer. This observation was concordant with the location of typical RT fields and with the fact that the highest doses are likely to be delivered to the anterior heart, including the LAD. As a consequence, MHD may be a poor surrogate parameter to reflect the dose to the cardiac sub-structures especially the LAD [11] and MHD may thus be a poorly relevant dose criterion for RT-induced cardiotoxicity studies and dose-response relationship.

However, a major issue is that coronary arteries are difficult structures to delineate because of their small volumes. Some atlas and auto-segmentation methodologies were developed for contouring cardiac substructures [12, 13], but they presented limits due to uncertainties for the smallest heart structures that are the coronary arteries. Another approach for coronary arteries’ radiation doses was presented in a previous work performed by Moignier et al. [14] where radiotherapy simulation CT scans and coronary computed tomography angiography (CCTA) of patients treated for a mediastinal Hodgkin lymphoma were used to merge thoracic and detailed cardiovascular anatomies and allowed personalized coronary arteries dose calculations.

In 2015, we launched the BACCARAT study, a cohort of a hundred of breast cancer patients treated with 3D-CRT and followed prospectively for 2 years with repeated cardiac imaging examinations including echocardiography and CCTA and measurement of circulating biomarkers. BACCARAT study aims to enhance knowledge on detection and prediction of early subclinical cardiac dysfunction and lesions induced by breast RT and on biological mechanisms potentially involved, based on functional and anatomical cardiac imaging combined with simultaneous assessment of multiple circulating biomarkers and accurate heart dosimetry.

With the rare opportunity given by the BACCARAT study to have simultaneous dosimetric and precise anatomical information from the simulation CT and CCTA images for all patients, the aim of this dosimetric study was to analyze the distribution of personalized individually-determined radiation doses to the heart and its substructures, in particular the coronary arteries, and evaluate whether MHD is a relevant surrogate parameter for the dose to cardiac substructures, in particular the LAD.

## Material and methods

The BACCARAT study, a monocentric prospective cohort study further detailed in a previous article [15], consisted in the inclusion of 114 women treated with adjuvant 3D-CRT for left or right unilateral breast cancer in the Clinic Pasteur in Toulouse, France, without chemotherapy, and followed for 2 years after RT (ClinicalTrials.gov: NCT02605512). Inclusion period lasted from October 2015 to December 2017. Follow-up of patients is still ongoing, the end is foreseen for early 2020.

After the surgical treatment of breast cancer, all patients were treated with 3D-CRT with 6 and 25 MV photon beams by tangential fields. Patients underwent planning CT scan, not contrast enhanced, with CT slices every 1.25 mm.

Patients were planned for RT with or without irradiation of supraclavicular or internal mammary lymph nodes. The planning target volume dose was 50 Gy delivered in 5 weeks with 25 daily doses of 2 Gy or 47 Gy delivered in 5 weeks with 20 daily doses of 2.35Gy. Additional boost of 9–15 Gy could be applied to the tumor site with electron/photons beams with energies ranging from 6 MeV to 18 MeV. Patients were positioned on a breast board with both arms above the head. The treatment planning system (TPS) used to perform dose calculations was Eclipse™ with the Analytical Anisotropic Algorithm (AAA v13.6) (Varian Medical System, Palo Alto, CA, USA). Each patient’s radiotherapy was planned such that the dose distribution was optimized and normalized to the International Commission on Radiation Units and Measurements (ICRU) reference point of the breast. Breath-hold gating was used for patients treated for left-sided breast cancer patients with heart very close to the anterior chest wall and for dose constraints achievement (Quantitative Analysis of Normal Tissue Effects in the Clinic - QUANTEC recommends keeping the volume of heart receiving at least 25 Gy (V25) less than 10% to keep the risk of cardiac mortality under 1% [16]; in addition, Clinique Pasteur attempts to keep MHD < 5 Gy). Dose-Volume-Histogram (DVH) for the heart was generated by the Clinic Pasteur radiotherapy department.

**Fig. 1:**
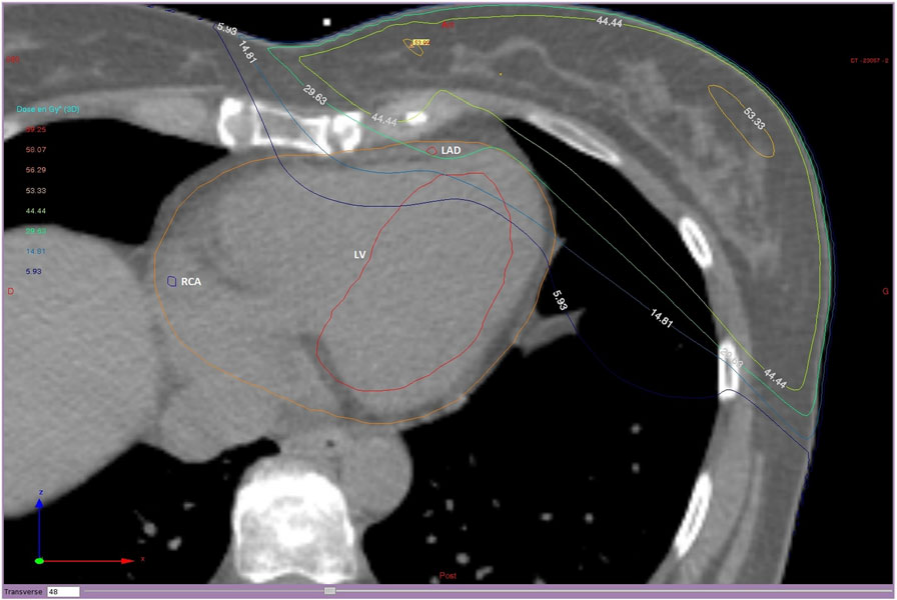
CT dose-planned left tangential breast irradiation showing isodoses and delineated structures: Heart (orange circle), left ventricle (LV), Left anterior descending coronary artery (LAD) and right coronary artery (RCA)

Before RT, a coronary computed tomography angiography (CCTA) was performed for all patients as planned in the BACCARAT protocol. One of the challenges of the BACCARAT study was to evaluate precisely absorbed doses to cardiac substructures, in particular focused on coronary arteries in order to enhance in a second step the dose-response analysis when considering early cardiotoxicity. The coronary arteries are narrow and moving structures; consequently the simulation CT scan makes proper contouring difficult or impossible. For dosimetric evaluation, the simulation CT scan, the CCTA, the RT dose and RT structure files in DICOM format were used as done in a previous study [14] . Merging anatomical information from the simulation CT scan and the CCTA was performed. The CCTA provided an optimal coronary visualization but at a given moment of the cardiac cycle, generally the diastole. On the contrary, the simulation CT scan provided images integrating the breathing and heart beating motion. Consequently, a slight rotation of the heart around the cranio-caudal axis, a homothetic deformation along the three dimensions and a translation to match the coronary arteries origins were carried out for the registration. Once inserted in ISOgray TPS (version 4.2, Dosisoft, Cachan, France; http://www.dosisoft.com/en/radiotherapy/planning-products.html), manual delineation was performed for the left ventricle (LV), the left main coronary artery (LMCA), the left anterior descending artery (LAD), the left circumflex artery (LCX) and the right coronary artery (RCA). Using the 3D dose matrix generated during treatment planning and the new delineated substructures, DVH for LV and coronary arteries were generated with ISOgray TPS by the dosimetric department of IRSN in collaboration with the Clinic Pasteur radiotherapy department (Fig. 1).

From the DVHs, the following absorbed dose metrics for all delineated cardiac structures were calculated: Dmean (in Gy) is the volume-weighted mean dose; D2 (in Gy) is the minimal dose received by the most irradiated 2% of the structure volume, which can be considered as the near maximum dose; V5 (in %) is the relative volume exposed to at least 5 Gy (similar definition for V10, V25 etc.)

Descriptive analysis of the physical doses in Gray (Gy) was performed. Continuous variables are presented with mean, standard deviation, median and range values. Categorical values are presented with percentages. The chi-square test was used to compare categorical variables and Student’s *t*-test or Wilcoxon non-parametric test if necessary was used to compare continuous variables. We defined individual ratio D2/Dmean as a kind of Homogeneity Index (HI) adapted to organ at risk as HI are usually used for planning target volume. Pearson’s correlation coefficients were used for correlation analysis between MHD and mean doses to the different substructures. The relationship analysis between MHD and mean doses to the different substructures were further investigated based on linear regressions providing the R^2^ value which corresponded to the coefficient of determination indicating the proportion of the variance in Dmean of the substructure predictable from MHD. R^2^ < 0.70 was considered insufficient for prediction and surrogate parameter purpose. *P* < 0.05 was considered statistically significant. All statistical analysis was performed with SAS software V9.2.

## Results

Retrospective dosimetric evaluation for all cardiac substructures was available for 104 BC patients (89 left-sided and 15 right-sided) from the BACCARAT study (Table 1). Mean age of the population was 58 years old, with no significant difference between right-sided and left-sided BC patients. Most patients (85/104) were diagnosed with an invasive ductal carcinoma and underwent breast conserving surgery. The prescribed radiation dose was 50 Gy in 25 sessions for more than three forth of the population. An additional boost of 9–15 Gy was applied to the site of the tumor if clinically indicated. Regional lymph nodes irradiation was performed in 31 left-sided BC patients and 1 right-sided BC patient.

**Table 1:** Patients baseline characteristics

	Left-sided BC patients *N* = 89	Right-sided BC patients *N* = 15
Age (in years)	56.0 ± 8.34 (40–74)	58.3 ± 5.8 (52–67)
Type of cancer		
In situ	16 (18%)	3 (20%)
Invasive	73 (82%)	12 (80%)
Tumor size (in mm)	12.7 (4–100)	8.3 (4–13)
Grade		
1	37 (41%)	7 (47%)
2	42 (47%)	7 (47%)
3	10 (11%)	1 (6%)
Surgery		
Breast conserving	82 (92%)	15 (100%)
Mastectomy	7 (8%)	0 (0%)
Prescribed dose		
50 Gy	68 (76%)	13 (87%)
47 Gy	21 (24%)	2 (13%)
Boost	81 (91%)	14 (93%)
9Gy	0 (0%)	1 (6%)
12Gy	66 (73%)	4 (27%)
12.5 Gy	13 (14%)	9 (60%)
15Gy	2 (2%)	0
Regional lymph nodes irradiation		
Supraclavicular alone	1 (1%)	0
Internal mammary alone	3 (3%)	0
Both	27 (30%)	1 (7%)

Results for each dose metric for the heart and its sub-structures are shown in Table 2. The mean MHD was 2.95 ± 1.49 Gy for left-sided RT, and 0.46 ± 0.12 Gy for right-sided RT. All patient met the dose constraint of the heart (V25Gy < 10%) with a maximum V25 value of 8.7%. The inter-patient variability in MHD was important especially for left-sided RT with a range of values from 0.87 to 6.72 Gy, further confirmed by the range in near-maximum dose D2 from 3.95 to 48.87 Gy. The intra-patient variability for heart doses was also extremely high, with average individual ratio D2/MHD of 8.4 for left-sided patient and 4.6 for right-sided BC patients illustrating the heterogeneity of doses within whole heart structure. Considering the other cardiac structures, LV and LAD were the most exposed structures with respectively mean doses of 6.2 Gy and 15.7 Gy (as illustrated for a patient in Fig. 1), but intra-patient variability in doses (D2/Dmean) was lower than observed for the whole heart (5.7 and 2.6 respectively). For the RCA, we observed that the mean absorbed dose was higher for right irradiation than left one (0.74 ± 0.53 Gy vs. 1.25 ± 0.51 Gy), and higher than MHD for right-sided BC.

**Table 2:** Dosimetric parameters for heart and cardiac substructures including coronary arteries

	Left-sided BC patients *N* = 89		Right-sided BC patients *N* = 15	
	Mean ± SD	Range	Mean ± SD	Range
Heart				
MHD (Gy)	2.95 ± 1.49	0.87–6.72	0.46 ± 0.12	0.25–0.67
D2 (Gy)	26.80 ± 17.40	3.95–48.87	2.16 ± 0.90	1.11–3.57
Individual ratio D2/MHD	8.4 ± 3.6	2.9–15.4	4.6 ± 0.6	3.6–5.8
Left Ventricle				
Dmean (Gy)	6.25 ± 3.53	1.16–13.42	0.09 ± 0.04	0.04–0.17
D2 (Gy)	34.01 ± 16.93	4.49–55.48	0.34 ± 0.13	0.17–0.54
Individual ratio D2/Dmean	5.7 ± 2.0	2.6–11.0	3.9 ± 0.7	2.6–5.3
Left Anterior Descending Artery				
Dmean (Gy)	15.68 ± 8.13	1.68–37.61	0.37 ± 0.15	0.13–0.54
D2 (Gy)	37.97 ± 15.93	2.19–56.42	0.45 ± 0.17	0.19–0.61
Individual ratio D2/Dmean	2.6 ± 0.9	1.3–6.0	2.3 ± 0.6	1.3–3.2
Left Circumflex Artery				
Dmean (Gy)	1.61 ± 0.83	0.53–4.34	0.14 ± 0.08	0.04–0.25
D2 (Gy)	1.87 (median)	0.75–48.66	0.27 ± 0.13	0.09–0.45
Individual ratio D2/Dmean	1.3 (median)	0.8–43.4	2.2 ± 0.8	1.5–4.0
Left Main Coronary Artery				
Dmean (Gy)	1.28 ± 0.69	0.60–4.72	0.11 ± 0.05	0.04–0.21
D2 (Gy)	1.39 (median)	0.69–49.45	0.25 ± 0.13	0.06–0.46
Individual ratio D2/Dmean	1.2 (median)	1.0–35.6	1.2 ± 0.2	0.8–1.6
Right Coronary Artery				
Dmean (Gy)	0.74 ± 0.53	0.14–4.39	1.25 ± 0.41	0.68–2.01
D2 (Gy)	1.38 ± 1.04	0.35–8.61	1.77 ± 0.74	0.33–3.54
Individual ratio D2/Dmean	1.9 ± 0.4	1.1–2.9	1.4 ± 0.4	0.3–2.2

*BC* breast cancer, *SD* standard deviation, *MHD* Mean heart dose, *D2* minimal dose received by the most irradiated 2% of the structure volume, *Dmean* mean dose to the structure

**Fig. 2:**
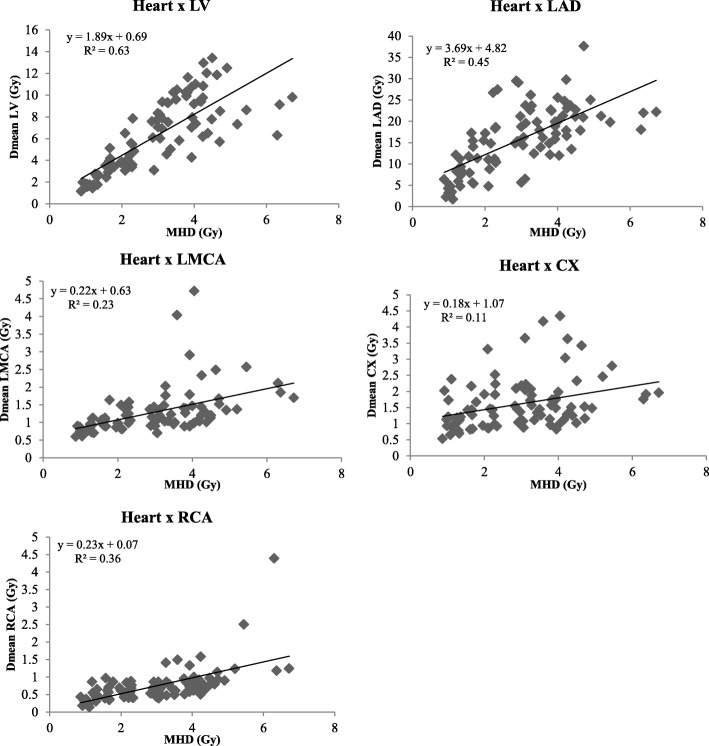
Relationship between MHD and mean doses to left ventricle and coronary arteries for left-sided BC patients

**Fig. 3:**
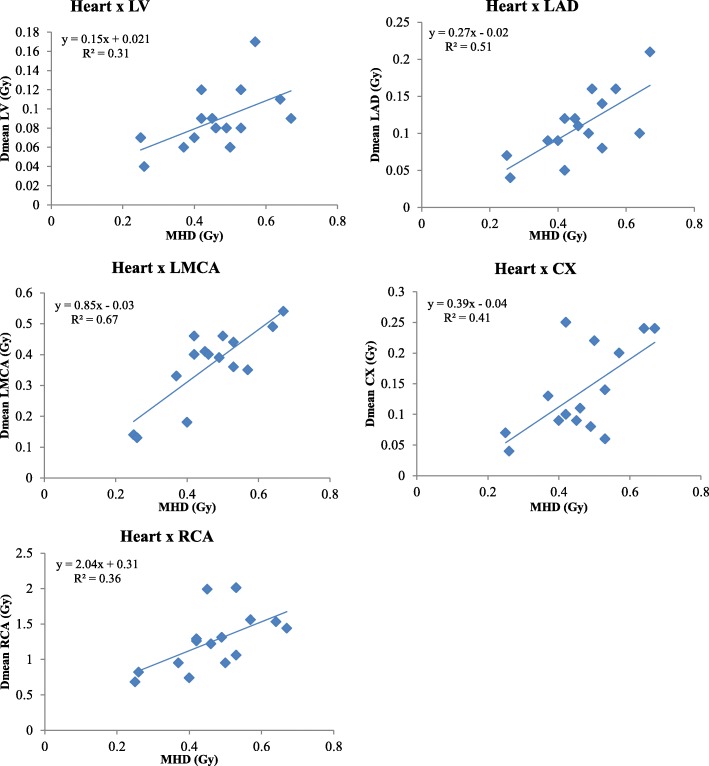
Relationship between MHD and mean doses to left ventricle and coronary arteries for right-sided BC patients

The ratios of Dmean for delineated structure and MHD are presented in Table 3. It allowed observing that for left-sided BC patients, the mean ratio Dmean LAD/MHD was around 5 and around 2 for Dmean LV/MHD. All other ratios were below 1 except for RCA in right-sided BC patients (2.7 ± 0.7). The strongest correlation with MHD was observed for LV (r = 0.78) and LAD (r = 0.67) for left-sided patients, and for LMCA and LAD (r = 0.81 and 0.71 respectively) for right-sided BC patients. Linear regression between Dmean for delineated structure and MHD are presented in Figs. 2 and 3. For every increase in MHD among left-sided patients, the Dmean LV and Dmean LAD increased on average 1.9 Gy and 3.7Gy respectively. However, the coefficients of determination R^2^ values indicated that the proportion of the variance in Dmean LV or LAD predictable from MHD was moderate for LV (R^2^ = 0.63) to low for LAD (R^2^ = 0.45). For every increase in MHD among right-sided BC patients, the Dmean RCA increased on average 2.0 Gy, but again R^2^ remained low (R^2^ = 0.36). For all others structures the relationship was moderate to weak. With no R^2^ value above 0.70, the predictive value of the MHD was not good for cardiac substructures including coronary arteries.

**Table 3:** Association parameters between mean dose to the left ventricle and coronary arteries and the mean heart dose

	LV	LAD	LCX	LMCA	RCA
Left-sided BC patients					
Ratio Dmean struc/MHD (mean ± SD)	2.1 ± 0.6	5.6 ± 2.2	0.7 ± 0.4	0.5 ± 0.2	0.3 ± 0.1
Correlation with MHD (r, *p-value*)	0.78, *< 0.0001*	0.67, *< 0.0001*	0.33, *0.017*	0.47, *< 0.0001*	0.60, *< 0.0001*
Right-sided BC patients					
Ratio Dmean struc/MHD (mean ± SD)	0.2 ± 0.1	0.2 ± 0.1	0.3 ± 0.1	0.8 ± 0.2	2.7 ± 0.7
Correlation with MHD (r, p-value)	0.55, *0.03*	0.71, *0.0026*	0.64, *0.01*	0.81, *0.0002*	0.60, *0.02*

*LV* Left Ventricle, *LAD* Left Anterior Descending Artery, *LCX* Left Circumflex Artery, *LMCA* Left Main Coronary Artery, *RCA* Right Coronary Artery, *BC* breast cancer, *SD* standard deviation, *MHD* Mean heart dose, *Dmean struc* mean dose absorbed by the delineated substructure

Considering the left sided BC RT patients, even in the “low exposed” group (MHD < 3Gy) high exposure to the LV and LAD was observed, D2 value that could reach 55.48Gy and 56.42Gy, higher than in the group of patient with MHD > 3Gy (Table 4). Moreover, in the “low exposed” group, 56% of the population could receive doses above 40 Gy to the LAD (75% in the high exposed group) that could reach 51.6% of the LAD volume, and 3 patients even received doses above 50Gy to the LAD with volumes of LAD of 1.2, 5.3 and 24.1% for these 3 patients.

**Table 4:** Highest doses to Left Ventricle and Left Anterior Descending Artery according to low or high MHD among left-sided BC patients

Left-sided BC patients	MHD ≤ 3Gy *N* = 45	MHD > 3Gy *N* = 44
Left Ventricle		
D2 (Gy): mean (range)	25.75 (4.49–55.48)	42.25 (21.30–53.17)
patients with V40 > 0	30 (67%)	34 (77%)
maximum value of V40	9.6%	20.0%
patients with V50 > 0	3 (7%)	14 (32%)
maximum value of V50	6.6%	7.0%
Left Anterior Descending Artery		
D2 (Gy): mean (range)	31.99 (2.19–56.42)	44.08 (18.60–53.32)
patients with V40 > 0	25 (56%)	33 (75%)
maximum value of V40	51.6%	59.5%
patients with V50 > 0	3 (7%)	8 (18%)
maximum value of V50	24.1%	50.7%

*MHD* mean heart dose, *D2* minimal dose received by the most irradiated 2% of the structure volume, V40 (in %) is the relative volume exposed to at least 40 Gy

## Discussion

Although there was considerable decrease in doses to the heart over past few years [8, 17, 18], radiation induced heart disease is still a concern due to the improvement in breast cancer patients survival. Mean heart dose was often used as the reference measure for cardiotoxicity studies [5]. However, there is increasing evidence toward the importance to consider individual cardiac substructures as some studies have implicated the left anterior descending artery [10, 19], as well as the left ventricle [7] as important components of the heart associated with radiation induced heart disease.

Our study highlighted that MHD was not representative of order of magnitude of mean dose to the left ventricle or even more to the most exposed coronary, i.e. LAD. The mean LAD dose (15.6Gy) observed among 89 left-sided BC patients was substantially higher than the mean heart dose (2.9 Gy) as previously observed [20]. Inter-individual variability in LAD exposure dose, illustrated by the wide range of LAD dose from 1.68Gy to 37.60 Gy, could be explained by several factors: - variation in heart size (larger heart tend to push the LAD into the radiation field), − variation in breast size (it modifies the extend of the tangential field), − individual coronary topology (coronaries are more or less tortuous [9], and in this study some were delineated longer than others because of intrinsic limitation of the CCTA imaging), − presence or not of boost and regional lymph node irradiation. The difference between LAD dose and MHD is striking, but it can be explained by the fact that the LAD is located in the anterior region of the heart where it is exposed to the tangential fields used in left sided breast RT. Moreover, we observed a mean ratio Dmean LAD/MHD around 5, confirming prior findings [21]. However, the association between the MHD and delineated substructures, in particular the LAD mean dose, was not precise enough to predict with confidence individual patient dose as shown in Fig. 2 and previously observed [22]. Despite significant correlation as observed in Table 3, this analysis showed that the predictive value of the MHD was not good for cardiac substructures including coronary arteries, even for LAD. More than 55% of patients with mean heart dose<3Gy could still be exposed to 40Gy or more to a part of LAD as illustrated in Table 4. In Darby’s study [5], estimated mean doses to the LAD and MHD were also correlated (correlation coefficient, 0.76 vs. 0.67 in our study) using a methodology based on a computed tomography of a patient with typical anatomy due to the period of inclusion of patients in a pre-computed tomography era. Such non individually-determined method could have led to inaccurate LAD dose estimations due to difficulties in localizing this structure in contrast with our method based on individual CT and CCTA. This could explain that the MHD was a better predictor of the rate of major coronary events than the mean dose to the LAD and even more surprising that inclusion of the mean LAD dose to MHD failed to improve the prediction of major coronary events rates as coronary arteries, in particular LAD, are critical to the subsequent development of radiation-induced cardiac diseases. In a more recent study [7], dose to the left ventricle (V5 precisely) was a better predictor of major coronary events than MHD. Similarly, in our study, the left ventricle was chosen as a structure of interest since its dose may be more representative of cardiac endpoints than the MHD. Indeed, left ventricle pumps blood into the systemic circulation to most of the body through the aorta (while the right ventricle fills only the lungs). The most common reason for referral to echocardiography is left ventricular function. Assessment of left ventricular function is extremely important as it correlates with symptoms, prognosis, events, and complications in a large number of conditions and many decisions in cardiology are based on left ventricular function [23] . From a dosimetry point of view, the association between the MHD and the LV dose as illustrated in Fig. 1 showed that MHD also failed in predicting with confidence individual LV dose.

In the cardiotoxicity assessment of breast cancer RT, dosimetric evaluation for RCA was poorly studied. However, RCA, is the second largest artery, after left main coronary artery, supplying the heart. During breast cancer treatment of right breast the proximal part of RCA can be included in the irradiation field. In our study we observed among right sided BC patients that mean heart dose was 0.46Gy but mean RCA dose was 1.25Gy. These results were concordant with those previously published [24] with a mean dose to the RCA of 1.92 Gy. The mean ratio Dmean RCA/Dmean Heart was around 3 and to a certain extend the RCA of right irradiated patients may play the same role than LAD for left irradiated patients.

Because of their small volumes and inherent contouring variability, the coronary arteries (in particular the LAD) are difficult structures to delineate. Dreaming of a ‘Swiss Army Knife’ for cardiac doses, some studies developed methodology based on atlas and auto-segmentation that could help to standardize contouring for these structures [12, 13, 25, 26]. These algorithms were able to generate valid segmentations for major cardiac structures (whole heart, Left ventricle, Right ventricle, …) but not for the small structures such as coronary arteries. In our study, rather than considering a generic coronary model based on anatomic charts or an atlas, we had the opportunity to perform an individually determined cardiac dosimetry including small cardiac substructures based on registration of the planning CT and CCTA images. However, a limitation of our study is that doses calculated by the TPS might not be reliable enough (out-of-field, lung/heart interface) but currently, it is what is available in the usual clinical context. Further investigations based on our data taking into account some of these dosimetric parameters based on heart DVH parameters and possibly other available clinical parameters, might lead to a better definition of the coronary dose, even if not delineated.

Very few previous studies have clearly defined the anatomic distribution of RT-associated coronary artery disease [14, 19]. One would reasonably hypothesize that the increased risk of coronary artery disease would be dose dependent and would manifest largely in the coronary arteries that are directly within the radiation field [27] as previously observed [19]. Our study allowed observing that the value of MHD could not help so much in understanding the dose effect relationship for the LAD. More research is needed to determine which indicator of heart dose from breast RT is the most significant for determining cardiac toxicity and morbidity. Doses to the heart has to be as low as possible. Actually, the QUANTEC - Quantitative Analysis of Normal Tissue Effects in the Clinic- recommendations, specify that the heart should always be contoured and that V25Gy < 10% (in 2 Gy per fraction) [16]. No dose recommendation exists for the coronary arteries, except one Danish study proposed a threshold with a maximum of 20 Gy to be given to the LAD [28] . With MHD as low as possible, the coronary arteries exposure could be as low as possible ad gating is an option to decrease the doses [22, 29] but we could not test it in our study as only 4 patients were concerned.

## Conclusion

To our knowledge it is the first study to use patient CCTA combined with patient-specific simulation CT scan to estimate dose to the substructures of the heart, including coronary arteries, in such a large number of patients. It allowed observing that taking into account the MHD only could nevertheless lead to excessive cardiac substructure irradiation and the predictive value of the MHD was not good for cardiac substructures including coronary arteries. The results of the study indicate that for precise radiotherapy-induced cardiotoxicity studies, it would be necessary to assess the dose delivered to the whole heart as well as to the cardiac substructures, in particular the LAD. For our BACCARAT study, this accurate heart dosimetry should be fruitful in the analysis of precise myocardial dysfunction based on cardiac echography and for precise analysis of coronary artery segments based on CCTA.

## Abbreviations

3D-CRT: 3 dimensional conformal radiotherapy
BACCARAT: Breast Cancer and Cardiotoxicity Induced by Radiotherapy
BC: Breast Cancer
CCTA: Coronary computed tomography angiography
CT: Computed tomography
DVH: Dose Volume Histogram
LAD: Left anterior descending coronary artery
LCX: Left circumflex artery
LMCA: Left main coronary artery
LV: Left ventricle
MHD: Mean heart dose
RCA: Right coronary artery
RT: Radiotherapy
TPS: Treatment Planning system
